# Augmented Reality–Assisted Training Tool for Mental Health Task-Sharers: Pilot Mixed Methods Usability Study

**DOI:** 10.2196/80711

**Published:** 2026-06-25

**Authors:** Ling Li Vivian Ngiam, Lucas Piotr Wozniak, Catherine Dinh-Le, Ayanna Elon Seals, Victoria K Ngo, Jose Fernando Florez-Arango

**Affiliations:** 1 Neurohue, LLC, 600 N Broad Street, Suite 5 #3720, Middletown, DE, 19709, United States, 1 3013668555; 2 Graduate School of Public Health and Health Policy, CUNY, New York, NY, United States; 3 Department Population Health Sciences, Weill Cornell Medicine, New York, NY, United States; 4 Center of Biomedical Informatics and Biostatistics (CB2), University of Arizona, 1230 North Cherry Avenue, PO Box 210242, Tuscon, AZ, 85721, United States, +1 (979) 481-7392

**Keywords:** augmented reality, augmented reality glasses, usability, task-sharing, participatory design, mental health care training, role-playing practice, soft skills training, simulation-based training, VP simulation, virtual standardized patient

## Abstract

**Background:**

The growing global mental health (MH) burden, especially in underresourced communities, calls for innovative, scalable, and culturally responsive training approaches to expand care access and improve outcomes. Task-sharing has shown promise in addressing workforce shortages but is limited by training and supervision challenges. Traditional methods, such as role-playing and standardized patients, are resource-intensive and less scalable. Virtual simulations, including augmented reality (AR), present novel opportunities for immersive and interactive training. An AR-assisted training tool can enable culturally sensitive training while fostering empathy, communication skills, and confidence in handling nuanced MH scenarios. However, AR’s usability and effectiveness for MH task-sharing training remain underexplored.

**Objective:**

This study aimed to assess the usability of an AR-assisted MH task-sharing training tool that uses virtual patient (VP) simulation and evaluate its potential to enhance training. Additionally, we developed design recommendations for future related XR-assisted clinical training tools.

**Methods:**

We conducted a formative, explorative sequential mixed-methods usability study. A convenience sample of 5 MH trainees or workers (ages 18‐60 years; female: n=3, male: n=2; identifying as African American, Asian, and Hispanic) participated. Participants were recruited through a university-affiliated MH training program. The usability testing protocol included a semistructured prestudy interview, orientation to the AR headset (Magic Leap 2), a think-aloud user testing session, and a poststudy quantitative questionnaire and qualitative interview. Usability was assessed using a modified Post-Study System Usability Questionnaire (PSSUQ), which measures system usefulness, information quality, and interface quality. Data were analyzed using descriptive statistics and thematic analysis.

**Results:**

The AR simulation was positively received by participants, demonstrating above-average usability. The overall mean PSSUQ score was 3.46 (SD 1.71), with subscale scores for system usefulness (mean 3.46, SD 1.77), information quality (mean 3.76, SD 1.73), and interface quality (mean 2.83, SD 1.59). Thematic analysis highlighted high realism fostered trainees’ empathy toward the VP, while increasing immersion and interaction quality. Despite some hardware limitations and user discomfort that broke immersion, participants recognized the tool’s potential and usefulness for training in various MH scenarios. Based on these findings, we proposed design recommendations across environmental context, training structure, VP behavioral realism (body language, voice, eye movement, and technical hardware considerations).

**Conclusions:**

This pilot study is among the first to evaluate AR-based VP simulation for training lay MH task-sharers, filling the technology and population gaps of prior VR- and desktop-focused simulation research. Preliminary empirical usability evidence from a validated instrument (PSSUQ) demonstrates above-average usability, and findings informed design recommendations for future research. These findings suggest AR-based training could provide a less resource-intensive solution for realistic MH training practice in underresourced settings. Further research includes larger sample sizes for inferential analysis, comparison studies with traditional methods, and generalizing to other MH conditions.

## Introduction

### Background

#### Current Global Mental Health Needs

The worldwide shortage of providers to meet mental health (MH) needs calls for innovative solutions to expand access to care and improve patient outcomes. Depression is a leading cause of global disability; evidence shows that investment in screening and treatment yields substantial health and economic returns [1,2].

In underresourced communities, there are greater barriers to MH care, which calls for context-specific and creative solutions. One factor that may prevent people from seeking appropriate care is MH stigma, which has been found to be higher in racial and ethnic minority populations [3]. Minority groups, including Hispanic and Black communities, are disproportionately affected and less likely to receive treatment [4]. Those who do receive treatment often receive lower-quality services [5].

To address these specific barriers to MH care, there is a need for increasing the supply of providers that exercise cultural humility, as such approaches are more effective at addressing the MH needs of culturally heterogeneous populations [6]. Populations impacted by discrimination and racism often express mistrust of nonminority providers [7]. Addressing these MH care gaps includes expanding appropriate training in cultural competence and instilling humble approaches of care in existing providers [8]. Thus, innovative approaches to training additional providers are needed to meet the growing MH needs of underresourced communities.

#### Current MH Training Landscape

MH task-sharing is a promising approach for increasing culturally sensitive MH services, particularly in underresourced communities [9]. Task-sharing shifts certain MH services from specialists to less-trained providers [10]. Studies show that task-sharing simple treatments, such as problem-solving therapy and medication management, have been implemented effectively [11,12].

Training and supervision are essential for successful task-sharing, as highlighted by the World Health Organization [13]. Systematic and locally specific efforts are needed to enable intervention sites to design and monitor their own training and supervision [14]. Additionally, simpler therapies, such as behavioral activation delivered by more junior MH workers, have been found to be as effective as more complex therapies delivered by specialists in improving outcomes for patients with depression [15]. Task-sharing interventions are also strengthened by incorporating the services of providers with strong community ties, shared lived experiences, credibility, and nonstigmatizing attitudes [10]. By training these community members and providers who are usually already providing informal support, communities can build their capacity to provide trustworthy MH services.

Resolving challenges associated with supervision and training is essential to improving MH treatment. Although clinical supervision and role-playing are useful training techniques, the conflicting priorities of task-sharing trainees often make it difficult to maintain regular training schedules, hindering the effective implementation of these methods [16]. Additionally, less experienced personnel may struggle to identify and treat subtle symptoms, such as reading facial expressions in patients with severe schizophrenia [17]. This underscores the importance of realistic simulation and accessible, flexible training tools. Traditionally, realistic clinical scenarios have been provided by standardized patients (SPs), trained persons who simulate actual patients and their symptoms [18]. However, this approach is resource-intensive, requiring significant time, travel, financial cost, and supervision [19,20].

Virtual standardized patients are computer-generated, interactive agents designed to simulate clinical presentations, allowing for repeated practice of MH skills such as interviewing and counseling [21]. Because virtual standardized patients do not require the same level of real-time oversight from supervisors, they can alleviate the burden on trainers by enabling self-paced, independent learning.

#### AR, VR, and Virtual Simulation

Virtual reality (VR) is described as a fully immersive virtual environment, while augmented reality (AR) overlays virtual elements onto the real world [22,23]. AR can foster a strong sense of presence and embodiment, making it a powerful tool in educational and training settings [24,25]. According to research, AR-driven identification and transportation can meaningfully affect users’ behaviors, physiological reactions, and psychological perception [26-31].

Social cognitive and situated learning theories support VR’s potential for observation-based learning in realistic contexts [32]. VR’s immersive and interactive nature also enhances experiential learning, making it effective in training by fostering empathy and user-centered thinking [33]. VR enables high-presence interactions with virtual patients (VPs) and creates realistic training environments [34].

Experiential learning through AR and VR in MH improves users’ comprehension, memory retention, and the development of practical skills by allowing them to participate in interactive simulations that closely mimic real-life scenarios [35]. These simulations elicit emotional responses similar to those experienced in real-life situations, nurturing empathy and profound emotional understanding, and providing valuable educational opportunities [36]. Additionally, AR and VR allow learners to practice in realistic environments, developing key skills like procedural recall, communication, problem-solving, and coping strategies [37]. Furthermore, AR and VR have been shown to be interactive, engaging, and convenient, hence making them valuable tools for virtual simulations in MH training and task-sharing settings [38].

#### Landscape of VR Training

VR training is increasingly used in health care education for both technical and nontechnical skill development [39,40]. In nursing education, a review of virtual simulations found them to be as effective as, or more effective than, conventional methods, offering self-paced, economical, and space-efficient learning opportunities [41].

In MH nursing, VR simulations facilitate emotional connections between learners and VPs by presenting realistic, interactive scenarios [42]. While earlier studies used nonimmersive, desktop-based VR, immersive VR with head-mounted displays (HMDs) has shown greater efficacy due to its sensory immersion, which intensifies focus, presence, emotional engagement, and learning outcomes [43-48]. In a usability test, a 360-degree VR simulation portraying schizophrenia scenarios was found to be user-friendly, engaging, educationally relevant, and highly immersive [49]. Another study on VR simulation training for behavioral health anticipatory guidance and motivational interviewing found that VR significantly increased pediatric residents’ engagement and critical skill development by providing an immersive, accessible, and realistic but patient-free learning and practice environment, and reduced their anxiety and stress [50].

#### Landscape of AR Training

AR is easier to use and has fewer negative physical side effects than VR, including the “cybersickness” that is sometimes felt after long VR usage [51]. AR also increases knowledge transfer by including real-world context that VR does not have [52]. In the medical field, AR is gaining popularity as a tool for clinical training and patient education by enabling medical professionals to visually communicate information about new treatments, medication mechanisms, and surgical procedures [53-56]. AR’s applications in anatomical and physiological training are noted for their maturity, showing substantial promise for transforming medical education through innovative prototypes and teaching approaches [57]. An integrative review by Zhu et al [58] found that AR has the potential to improve health care education by lowering failure rates and enhancing precision, while other studies also highlight AR’s role in enhancing knowledge retention, concept integration, and confidence among medical students [58-62]. For example, a pilot study contrasting traditional mannequin-based training with AR-enhanced approaches for patient decompensation recognition demonstrated that AR significantly improved participants’ assessment skills and clinical confidence [63]. Despite promising early findings, there are still relatively few published experimental and observational studies assessing AR’s application and efficacy in medical education [23].

AR in medical education has shown promise in enhancing learning and practical skills, and it has further potential in providing immersive environments for the development of important social skills [64]. By superimposing virtual components within the real environment, AR is very suitable for effective simulation training [58]. Ward et al [65] expressed that exposing users to challenging or rare scenarios in AR simulations creates a safe environment for practicing adaptive skills needed in the clinical space. Despite AR’s potential to deliver intricate and precise training across diverse social skills applications, the literature remains lacking, especially within MH training. A recent systematic review pointed out that while simulation-based training in health care education is advancing, there is limited systematic research on its impact on learning outcomes, with most studies to date focusing on feasibility and face validity [64].

Using an AR tool for MH simulation training with VPs is novel; hence, usability testing, defined as the evaluation of elements that impact users’ experience with a product for its intended purpose, is an essential first step to determine the strengths, weaknesses, and effectiveness of using AR for task-sharing [66]. Thus, we conducted a mixed-methods pilot study assessing the usability of our AR tool.

### Research Objective

Building on the above existing research, we first engaged in a participatory design process to investigate the various needs and challenges pertaining to AR-assisted training of lay MH workers in conducting MH screening and assessments of community members. We then built a prototype which was pilot-tested with stakeholders from the Harlem Strong Initiative (HSI). The HSI, led by the CUNY Center for Innovation in Mental Health (CIMH) team, uses community-engaged planning across a multisectoral coalition of organizations to support MH task-sharing and evaluates the impact of this model in low-income housing and primary care sites in the Harlem community [67,68].

The goals of this study are to assess the usability of the AR-assisted MH task-sharing tool in the current training landscape and to propose design recommendations for future iterations of AR-assisted training tools for MH task-sharing skills and related use cases.

## Methods

### Research Design Overview

The study design used a formative mixed-methods approach incorporating both qualitative and quantitative data. This design triangulates objective usability scores with subjective user experiences. We chose a modified “think-aloud” protocol for user testing to gather insights into study participants’ cognitive processes and any usability difficulties they had during the experience [69]. As a group, we outlined questions for pre- and posttesting qualitative interviews grounded in literature, and we used the Post-Study System Usability Questionnaire (PSSUQ), which has prior validity evidence to assess usability quantitatively [70].

### Prototype

The prototype was co-designed with community leaders and members of the Harlem community. Methods used during the participatory design included interviews, field ethnography and observations, early user testing workshops, and multistakeholder co-design sessions.

To better understand and design for the community, we explored three research questions: first, what are the various needs and challenges pertaining to training community-based organization workers in conducting MH screenings of community members; second, how can an AR-assisted MH task-sharing training tool potentially address those needs and challenges; and third, what interaction design strategies can be used to improve the usability of such a tool.

Interviews and brainstorming workshops with community stakeholders highlighted key needs for the training tool- being time-efficient, offering engaging and realistic content mirroring the nuanced scenarios social workers often face, catering to diverse backgrounds, especially the African American and Hispanic communities, while accommodating varying levels of MH literacy, embedding emotional support for social workers during intense scenarios, and fostering a sense of self-efficacy.

During a field observation session of CIMH’s existing online training, screening skills were highlighted as a curriculum section that many trainees wanted to explore further. The Patient Health Questionnaire-4 (PHQ-4) form specifically offers a self-contained, well-structured, brief training scenario that requires soft skill expertise to administer effectively. It consists of four questions that assess symptoms of both anxiety and depression, frequently used for clinical assessments and research purposes [71]. Thus, administering the PHQ-4 was integrated into the design of the prototype.

**Figure 1.**
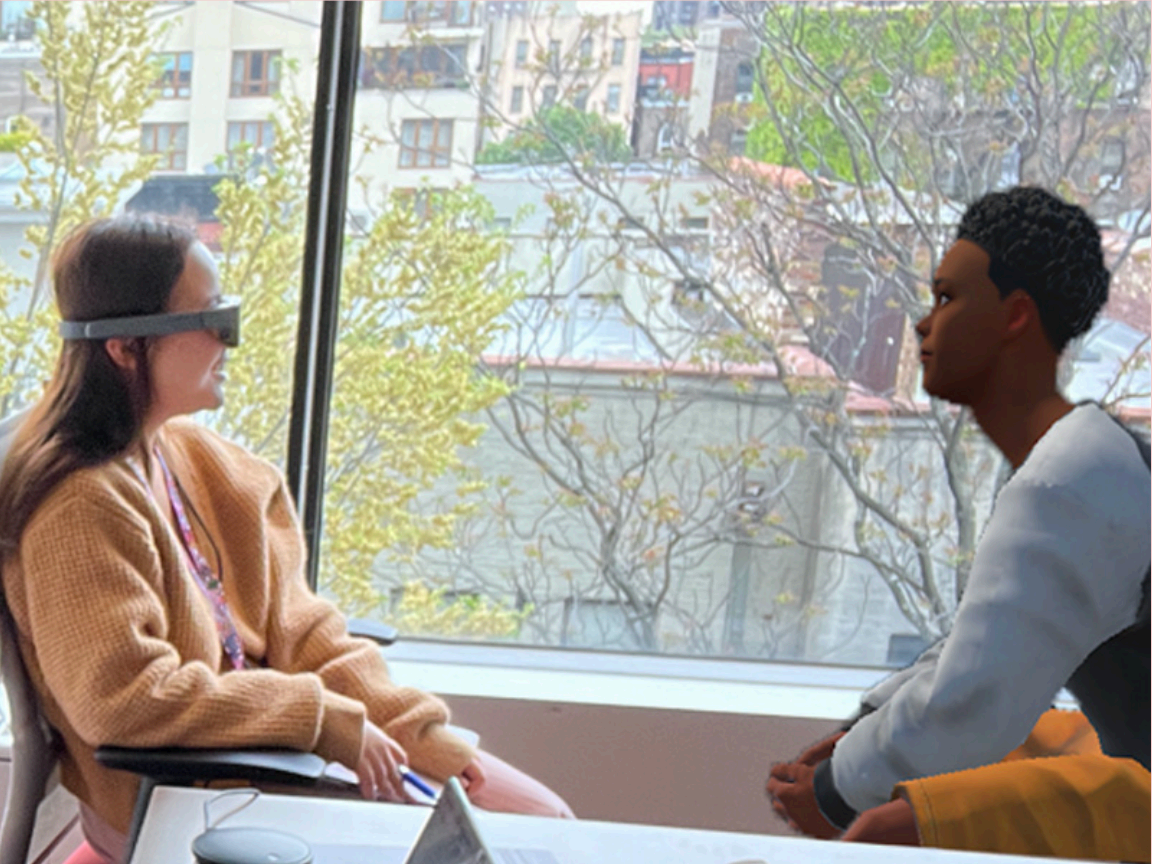
Profile view of a participant wearing the Magic Leap 2 augmented reality headset while interacting with the virtual patient training prototype. The virtual patient appears through the headset and is positioned across from the participant to simulate a face-to-face mental health screening and communication scenario.

After several co-design sessions, the prototype was finalized as a single MH screening skills module guiding trainees through administering the PHQ-4 appropriately (Multimedia Appendix 1). As seen in Figure 1, the user puts on the AR glasses, Magic Leap 2, and role-plays as an MH provider and interacts with a young adult, Hispanic, male VP named Alvaro, who exhibits symptoms of depression and hesitates about opening up about them [72].

**Figure 2.**
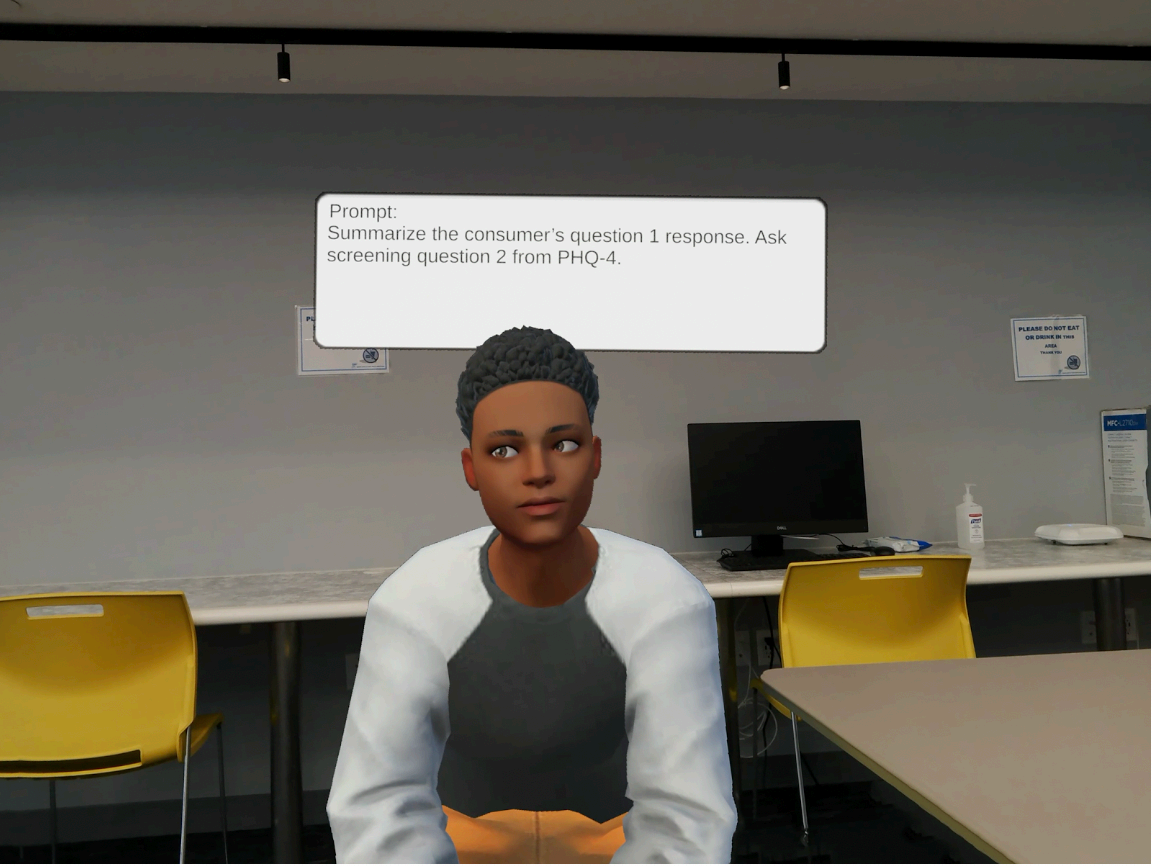
First-person headset view of the virtual patient embedded within the real-world lab environment. The virtual patient is designed to simulate conversational screening interactions and respond to user input during the augmented reality-based training experience. PHQ-4: Patient Health Questionnaire-4.

Trainees are guided by 24 different dialogue prompts (Multimedia Appendix 2) that appear above the VP in a roughly 10-minute simulated interaction (Figure 2).

**Figure 3.**
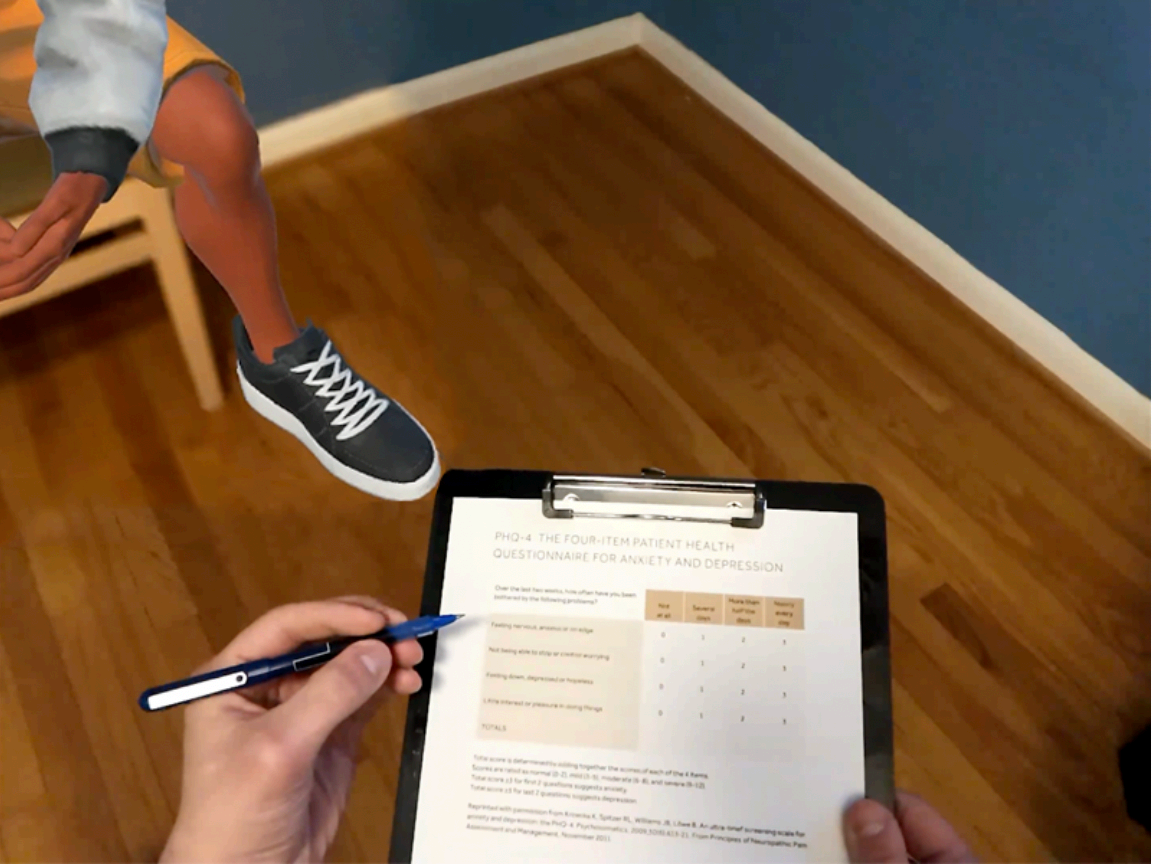
First-person view of the Patient Health Questionnaire-4 screening form presented during the simulation. Participants completed the standardized questionnaire as part of the virtual patient interaction workflow. PHQ-4: Patient Health Questionnaire-4.

The VP responds with human-recorded spatial audio voiceovers, lip-synced to animated facial expressions, as well as various body language animations, which enhance the realism and emotional aspect of the experience. Trainees can ask follow-up questions at times or ask the VP to repeat what they said. The experience allows them to practice new communication skills verbally and in an embodied way, engaging with the VP while filling out four responses on a physical PHQ-4 form (Figure 3).

### Data Collection

#### Participants

A convenience sample of participants was recruited from CIMH’s community network through listservs and word of mouth. Inclusion criteria were broad to capture a wide range of task-sharing perspectives, requiring that participants were members of or adjacent to the community served by the HSI and could speak to the needs of local MH training. Participants recruited for this study group did not overlap with study participants in the main HSI study. Education and MH training levels were varied to generalize the usability of the training tool; participants ranged from a college student with no MH training to an experienced licensed clinical social worker. While five participants were initially recruited and consented, one participant withdrew before beginning the user testing stage. Consequently, the final analytical sample consisted of four participants (n=4), representing a 20% proportion of missing data for the simulation and poststudy measures.

#### Usability Tests

There were 5 back-to-back usability tests scheduled in a day, with each session lasting between 45 minutes and 60 minutes. Participants were compensated with a US $50 Amazon gift code for their contributions. Their informed consent was first obtained through a physical form, after which we carried out a five-stage data collection procedure:

Participants first engaged in a semistructured prestudy interview, detailing their training background, including their experience with CUNY’s MH skills modules and their role in the community. They also discussed the challenges and strategies associated with interactions with distressed community members who could be experiencing MH issues. (Multimedia Appendix 3).

Participants were then briefed about the study goals and AR simulation objectives. They were provided with clear instructions about the AR glasses usability test, emphasizing the experience over their knowledge.

After an orientation to the AR glasses, participants navigated an AR simulation, practicing the administration of the PHQ-4 form with a VP. To facilitate the “think-aloud” protocol, researchers provided a structured briefing and used standardized verbal probes during the simulation. Participants were prompted to describe visual elements (eg, “Describe what you see”), instructed on how to handle interaction cards (eg, “Show us how you would proceed”), and asked targeted questions during planned pauses regarding the character’s body language and the use of the PHQ-4 task. If participants deviated from expected interactions, researchers asked clarifying questions such as “What did you expect to happen?” to capture their cognitive processes.

A semistructured poststudy interview guide was used to assess participants’ perceptions of the usability of the AR simulation (Multimedia Appendix 3). Interview guide questions were adapted from usability testing literature [73-75]. The interview aimed to query feedback on the prototype’s use, effectiveness, and potential improvement areas. At the end of each interview, the researcher confirmed all relevant information had been included to ensure the quality and accuracy of the data.

Finally, a 17-question poststudy questionnaire (Multimedia Appendix 4), adapted from the PSSUQ with a 7-point Likert scale ranging from strongly agree (1) to strongly disagree (7) with N/A as an additional option [70], was administered. The PSSUQ is a validated instrument with high internal consistency (typically Cronbach α>0.89), designed to measure user satisfaction across system usefulness, information quality, and interface quality. Additionally, demographic questions with multiple choice options were included that determined Table 1.

**Table 1.** Overview of interview participant characteristics, including age range, gender, ethnicity, prior augmented reality experience, and highest level of education. One enrolled participant withdrew prior to completing the study.

Participant ID	Age range (years)	Sex	Ethnicity	No. of times used an AR^a^ or similar device	Highest level of education
P1	35‐40	Male	Latino or Hispanic	0	Master’s degree
P2	26‐30	Female	Asian	1	Bachelor’s degree
P3	56‐60	Female	African American	0	Master’s degree
P4	16‐20	Female	Asian	0	High school
P5 (dropped out)^b^	—^c^	Male	African American	—	—

a AR: augmented reality.

b Participant wore glasses, went to get their contact lenses but never came back.

c Not available.

**Figure 4.**
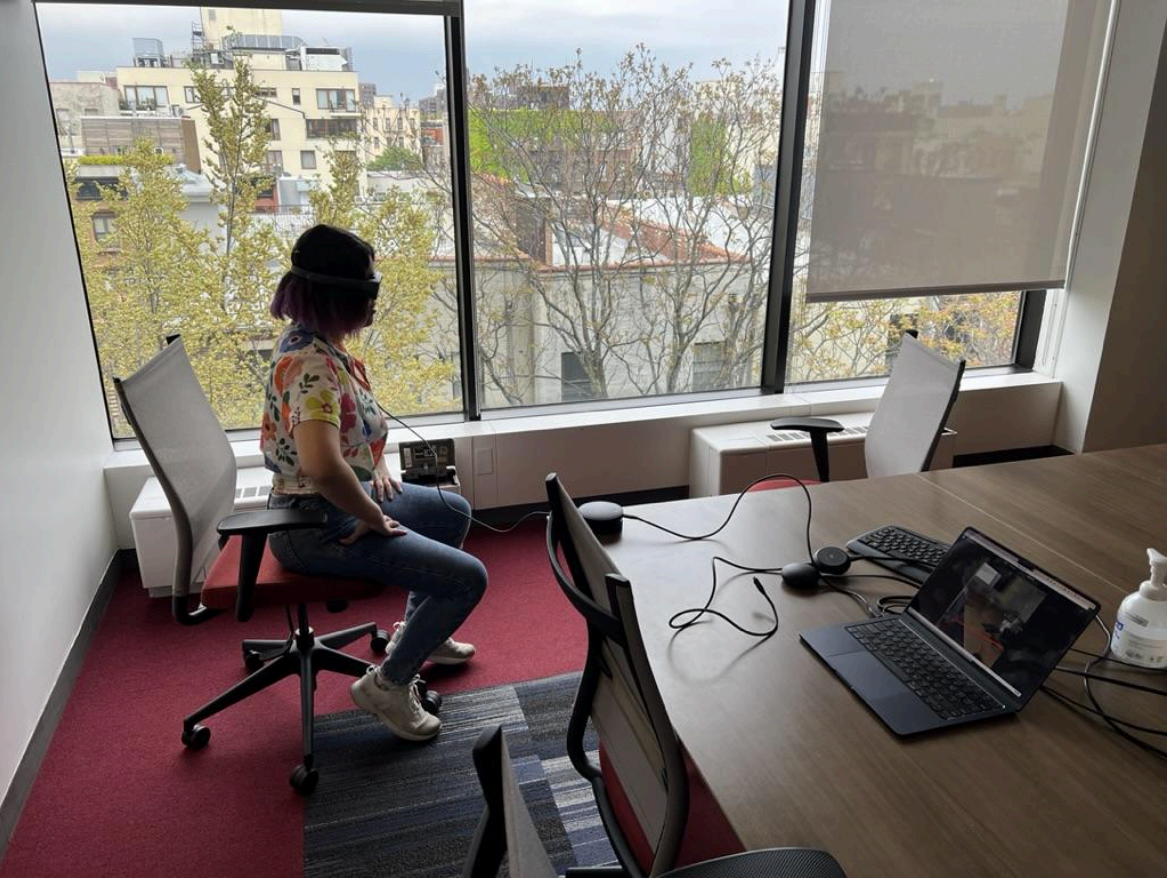
Individual participant setup during usability testing, showing the seated configuration used for augmented reality-based virtual patient interaction and observation.

**Figure 5.**
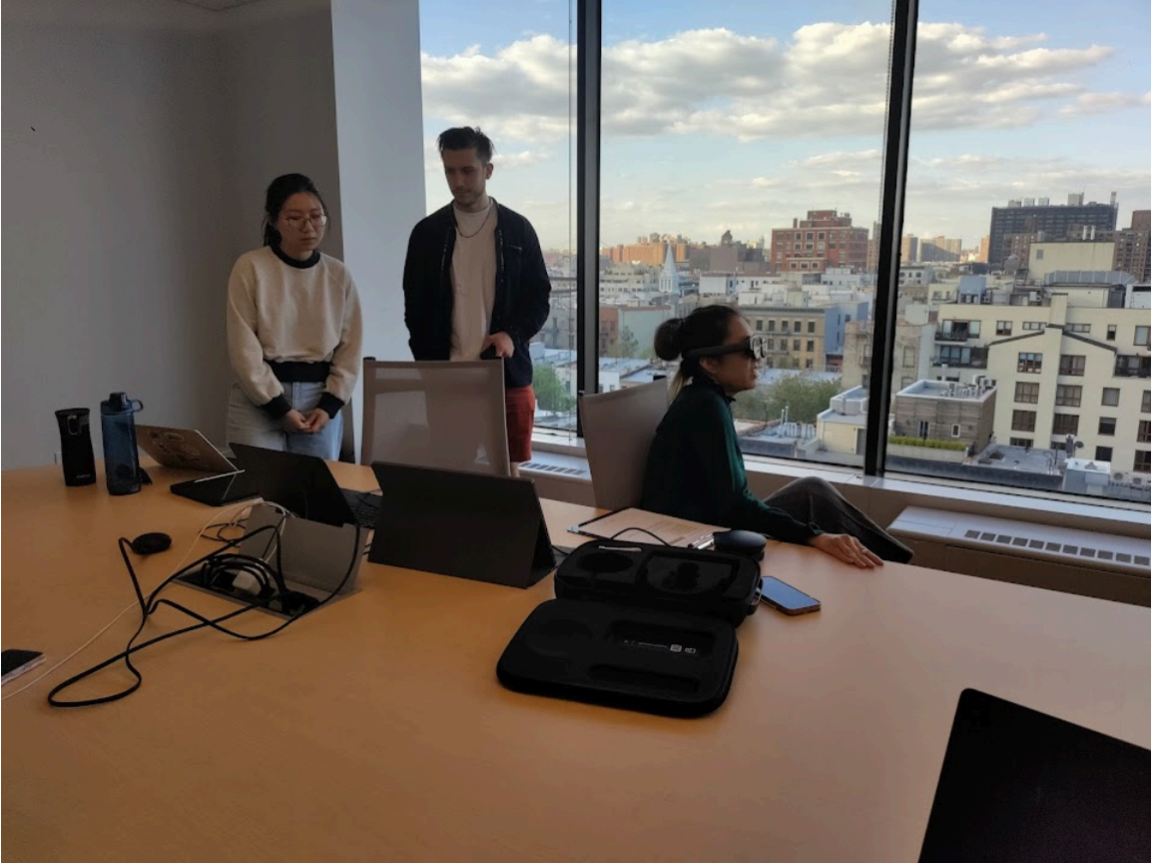
Usability lab setup including facilitator workstations and participant seating arrangement used during augmented reality training sessions.

The testing took place at one of CIMH’s facilities, in a large private room with several tables and chairs. The first and second authors conducted the usability tests and were the only people present in the space besides the study participant. The hardware used includes the Magic Leap 2 and two laptops for filling out the study questionnaires. The prototype was developed in Unity, using Ready Player Me for the custom avatar, Salsa LipSync Suite for facial animations, and Mixamo for body animations [76-79]. The usability lab featured two facing chairs (Figure 4), one for the participant and one for the VP. Researchers were positioned outside the participant’s field of view (Figure 5) and intervened only for technical issues or at specific checkpoints (Multimedia Appendix 3).

### Data Analysis

#### Quantitative

We used descriptive and summary statistics to analyze participant demographics and scores on the adapted PSSUQ. Due to the pilot nature of the study and the small sample size (n=4), inferential statistical testing was not performed; data are presented as mean (SDs) to indicate trends.

The PSSUQ consists of a set of 17 questions on a 7-point Likert-scale (1=strongly agree to 7=strongly disagree). The overall PSSUQ score is the average of the scores of questions 1 to 15 and 17. The subscales include: system usefulness (SYSUSE), which is the average score of questions 1 to 6, information quality (INFOQUAL), which is the average score of questions 7 to 12, and interface quality (INTERQUAL), which is the average score of questions 13 to 15.

#### Qualitative

All interviews were recorded using Zoom, with identifying details removed to maintain confidentiality [80]. Initial transcriptions, facilitated by Zoom’s automatic service, were refined by the first author, taking out any names, occupations, and other identifying details and using a prescribed participant number instead. After transcriptions were verified for accuracy, they were entered into the qualitative analysis software ATLAS.ti (ATLAS.ti Scientific Software Development GmbH), where a 2-step coding process was adopted [81].

Two independent coders (VN and SF) were used to minimize bias and positionality and ensure accurate depiction of participants’ perceptions [82]. The first author (VN) and a research assistant (SF) used an inductive approach based on conventional qualitative content analysis to analyze the interview data [83,84]. In contrast to summative content analysis, which focuses mostly on counting and measuring data, this method emphasizes the identification of recurrent patterns throughout the data collection, similar to traditional thematic analysis [85].

The researchers independently reviewed the interviews and subsequently coded key concepts in line with the interview questions while also drawing from codes from the participatory design phase, such as “Challenges,” “Workflow,” “Ease of use,” “Immersion,” “Usefulness,” and “Future recommendations.” Then, the researchers discussed preliminary findings since multiple analysts for code development enhance the findings’ credibility [82]. Using an open coding strategy, codes were refined and added, reflecting significant patterns in the data. Discrepancies in coding led to discussions until consensus was reached. The frameworks described in a related study by Hess et al [86] served as further guidance for the coding process. Domains from the study like “Experiential satisfaction,” “Learning engagement,” “Technology learning curve,” and “Opportunities for improvement” formed the basis for theme identification.

Transcripts were then restructured according to common codes for thorough examination of themes and patterns. Participants’ frequently overlapping and closely related codes were grouped together to create categories, which in turn led to the formation of primary themes. The categories were formed and summarized individually. Comparing these categories and summaries between the 2 researchers constantly and discussing to reach consensus allowed for principal themes (Multimedia Appendix 5) to be formed with a consistency of more than 90%, suggesting additional reviews were unnecessary [82].

### Ethical Considerations

This study was approved by CUNY’s Institutional Review Board (IRB2021-2031) as part of the Harlem Strong Mental Health Coalition study (U01OD033245). All procedures performed were in accordance with the ethical standards of the institutional and/or national research committee, and written informed consent was obtained from all participants. The consent forms included information on the study purpose, expected procedures, time commitment, potential risks, discomforts, and/or benefits, such as participation payment, and participant confidentiality and rights. Participants were given descriptions of how and with whom their data would be safeguarded, used, and shared; details in the consent form included the potential for deidentified data submission to the National Institute of Mental Health Data Archive (NDA) for secondary analysis without additional consent.

Authorized members of the research team managed the password-protected data for any identifiable information that might be linked to participants from this study. Codes were assigned to replace names of participants, and identifying information was removed when no longer needed. Participants were compensated with $50 Amazon gift cards after the completion of their session. In terms of included images of participants completing the study, only the study team members are identifiable in photos. Participants all wore the AR headset, and written permission was obtained to include their deidentified images.

### Validity, Reliability, and Methodological Integrity

In accordance with JARS-Qual standards, we acknowledge the influence of the research team’s background on data interpretation. The primary investigators include digital health informaticists, HCI researchers, and clinical psychologists with extensive experience in MH task-sharing. This diverse expertise allowed for a rigorous evaluation of the tool’s technical performance while remaining grounded in clinical reality.

Quantitative reliability was supported using the validated PSSUQ instrument. Qualitative integrity was maintained through investigator triangulation and the use of the think-aloud protocol, which captured real-time cognitive data, reducing retrospective recall bias.

## Results

### Overview

We recruited a total of 5 lay MH trainees and workers with various ranges of training and experience in MH care (for participant details, see Table 1).

### Prestudy Interview

To set some context for this inquiry, we present a brief background of relevant current workflows described by the pilot study’s participants during the prestudy interviews.

#### Building Trust Is Challenging but Important

Building trust with the community was highlighted as crucial by two participants. P1 told us that they

definitely use a lot of open-ended questions,

While P3 highlighted that

Trust is a huge factor in getting community members to know that you’re there for them, that you’re concerned about their well-being. You’d like to maybe introduce them to some practices that they may not be aware of or might not understand fully and let them know that it’s okay.

P3 further described:

I might tend to go a little bit further than intended for whatever the session is supposed to be, as more of a listening ear type thing, and just try to engage in trying to get more information from them that I might not necessarily use.

#### Difficulty in Adapting Theoretical Knowledge to Practice in Current Training

Participants experienced difficulty adapting theoretical knowledge to practice. P2 noted that

because every encounter is different, it’s kind of very foundational. You have to develop your own way of counseling,

While P1 emphasized that

when you come into actually working in the field, it’s a little different. You might be in a setting where you’re dealing with things that are the least favorable settings.

#### Current Training Included Role-Play and Usually Did Not Include Difficult Scenarios

P1 told us that in the current workflow they: *“covered* The *Diagnostic and Statistical Manual of Mental Disorders,*” which is a comprehensive classification system published by the American Psychiatric Association (APA), and *“did a lot of role-play.”* They added that *“normally, when we do role-plays in class, nobody goes down that road of re-enacting more challenging scenarios like schizophrenia, people tend to present the lighter stuff that might not be entirely accurate.”* This highlighted that current training included role-play and usually did not include difficult scenarios, which corroborated with our prototype design to focus on role-play.

#### Content of Sessions

P1 also noted that typical sessions were brief:

Most of the sessions that I work with were 20 min sessions. So if I only had 20 min to gauge how a person’s week went, you know, to go through anything larger than 5 questions to gauge their temperament will be challenging.

This informed our prototype design choice of administering the brief PHQ-4 form.

### Usability Findings

Overall, the four participants rated the usability of the prototype as above-average usability (Multimedia Appendix 6) in the modified PSSUQ survey which includes an additional question on the desired frequency to use the system (question 16).

**Figure 6.**
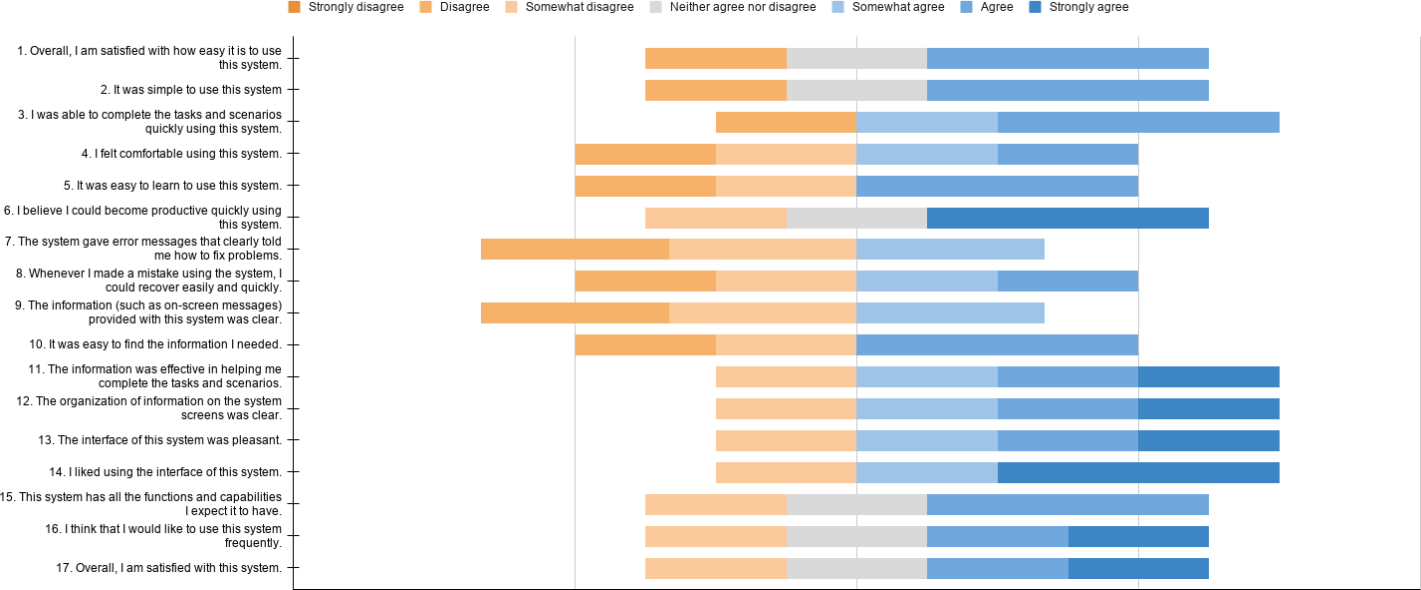
Distribution of participant responses to the Post-Study System Usability Questionnaire. Bars leaning toward agreement indicate positive perceptions of system usability. N/A responses were excluded from the visualization.

The prototype received an overall PSSUQ score of mean 3.46 (SD 1.71), SYSUSE score of mean 3.46 (SD 1.77), INFOQUAL score of mean 3.76 (SD 1.73), and INTERQUAL score of mean 2.83 (SD 1.59). Desire to use the system frequently (question 16) received a mean score of 3 (SD 1.83). Figure 6 displays the distribution of responses across all items. One participant chose N/A for questions 7 and 9, which skewed those results. Participants who identified as Asian experienced difficulty with the headset fitting comfortably on their faces (due to a missing nose piece kit) and gave higher scores on their PSSUQ questionnaire.

### Think-Aloud Insights

In this section, we outline the observations made during the think-aloud section in which participants interacted with the intervention.

#### Use of Physical Headset

Two out of the four participants experienced the headset falling off their face, requiring constant adjustment and even manual holding to continue. P2 mentioned that

it broke the immersion to me. I think it would feel more like I’m talking to someone if the headset didn’t keep falling, I was distracted by it.

P4 felt similarly:

it’s just slipping down everywhere. My arms were like sore from holding it up because they’re heavy, too. But yeah, I think I was a little bit distracted by how like it kept falling down. I think also like the whole thing with the headset not fitting just like took away from it.

By P4’s session, the headset was also heating up, compounding discomfort.

#### Delivery of Content

All participants reported difficulty hearing the VP’s audio at maximum volume, requiring researcher clarification at multiple points.

P3 and P4 also reported difficulty understanding the VP’s accented speech at times, with P4 unable to comprehend certain responses even after replays.

P2 and P4 experienced technical glitches where the VP failed to respond to prompts, requiring researcher intervention to continue.

### Key Themes From Postintervention Interviews

Following the think-aloud section, the study’s major themes identified from the postintervention interviews are discussed here, mainly experiential satisfaction, navigation of the training experience, and interacting with the VP.

#### Experiential Satisfaction

The overall impression of the simulation, particularly the emotional valence and level of satisfaction for the participants, was generally positive, consistent with the above-average overall PSSUQ usability score (mean 3.46, SD 1.71). The simulation’s realism allowed for participants to feel empathy toward the VP. Participants acknowledged the potential usefulness of the simulation in other contexts, and first-time AR and VR headset users also voiced some struggles.

##### General Positive Sentiments

Participants generally had positive sentiments toward the training module and found the simulation to be very useful. P2 expressed that “it’s a really good training module and the prompts were very helpful in guiding.” Echoing similar sentiments, P3 pointed out that this was their first AR and VR experience and that “it was cool and successful.” Drawing connections to current training, P1 mentioned that adding this kind of training and technology would be beneficial for practicing difficult cases during role-playing training as they could better depict challenging scenarios. They emphasized:

I think maybe having specific augmented reality trainings for specific DSM categories would be very helpful … like depression and anxiety … if it’s tailored towards, you know, something that may be heavier, that would be interesting.

Furthermore, they highlighted that the usage of the PHQ-4 form itself would be useful for training as it would also be helpful to use it in the field.

##### Realism Resulted in Empathy for the VP

The realism of the simulation and the quality of the animations led participants to feel empathy toward and connect with the VP. Participants all expressed that they felt like they were talking to a real person, and some also mentioned that they forgot that the researchers were in the same room while having the conversation, as the simulation felt so realistic. Three out of the four participants also noted that the voice of the VP felt very realistic as it had an accent, making the scenario very similar to what it would be like in the field. P1 emphasized that

it kind of forces you to empathize. It forced me to look at the AI almost like a real person, like, this person has an accent.

##### Potential Usefulness of Simulation in Other Contexts

Furthermore, participants highlighted the potential usefulness and applicability of the training approach in other contexts, especially in different MH and specific *DSM* (*Diagnostic and Statistical Manual of Mental Disorders*) categories. Other contexts mentioned include general one-on-one interactions, for example, in customer sales training. With regards to other MH contexts, P1 pointed out that it would be a lot more beneficial to carry out challenging role-play scenarios through AR than to act them out, as it may misrepresent symptoms of, for example, schizophrenia. P3 also highlighted that it would be extremely useful to have simulations like this used in the context of different MH conditions, as having and practicing the lived application of this knowledge is important for people who interact with people who might display these conditions.

##### Struggles for First-Time AR and VR Headset Users

Two out of the three first-time AR and VR headset users told us that they were slightly confused at the start when trying to navigate the experience. They felt a little overwhelmed between having to pay attention to the prompts, ask questions from the PHQ-4 form, and pay attention to the VP’s speech while trying to understand the accent, be attentive to the VP’s body language, and maintain eye contact. The uncertainty of what would come next initially also made the participants feel uneasy. On the other hand, P1 told us that

if this was my second or third practice, I would know I can read the prompt and look at the patient.

Two participants mentioned that with more practice, they would be able to get the hang of the training and reap the benefits from it.

### Navigating the Training

This section details the participants’ experience with the system’s guidance mechanisms, providing context for the information quality subscale (mean 3.76, SD 1.73), where the instructional design was found to be functional but occasionally confusing.

#### Effectiveness of Prompts

**Figure 7.**
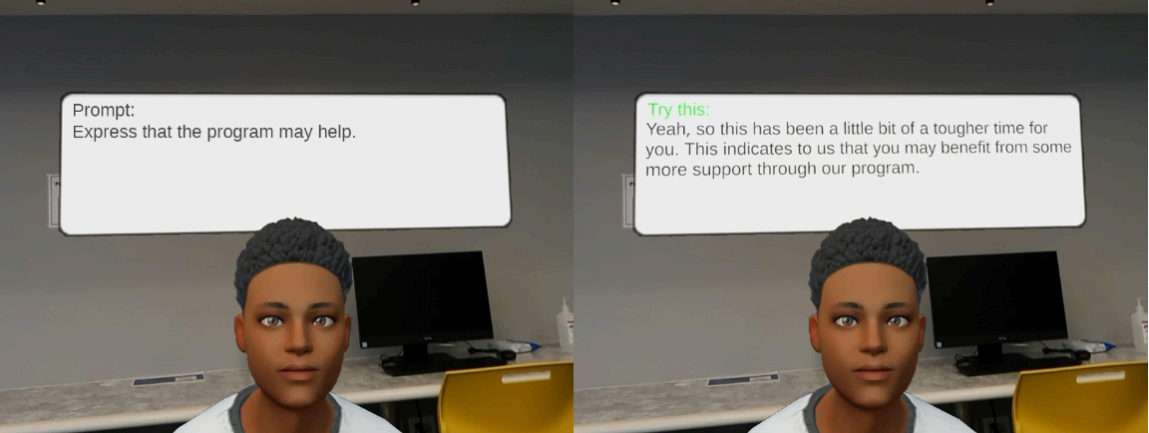
Comparison of two in-system prompt styles within the virtual patient simulation: (left) a general guidance prompt encouraging users to respond in their own words, and (right) a structured “Try this” prompt offering a suggested phrasing. The figure illustrates alternative scaffolding strategies tested for communication support.

Overall, participants found the prompts (Figure 7) useful in guiding them through the training. P3 appreciated that the prompts helped them focus on the specific goal at that certain time and that the prompts worked well in helping them follow the flow of the training. P4 mentioned how the prompts helped them to learn appropriate responses when they were unsure of how to respond. They told us:

I’m not sure if *I*m like you know, helping or hurting. So that’s why having the prompts helped. It’s like semantics also have impact on how you affect the client ...

They further mentioned that

… at times I was really lost as to what to say, but also because I didn’t hear him so well. That kind of made it more helpful for the prompt, because I was like, okay, this is what I say now.

However, navigating between 2 types of prompts (Figure 7) confused users and reduced immersion. Participants mentioned that having to pay more careful attention to the 2 different kinds of prompts broke the flow of immersion and took away from the experience. P2 elaborated that

when it’s the ‘Try this’ prompt, I’m like kind of fed what to say, but then there’s the other prompt where I kind of have to think on my feet. Switching back and forth was kind of confusing for me.

P3 and P1 similarly felt that the simultaneous demands of attending to VP dialogue, body language, and PHQ-4 questions alongside varying prompt types created cognitive overload. P1 wanted more attention to the VP’s “body languages, whatever it may be, tone of voice, things of that nature,” hence the navigating between the 2 prompts did sometimes confuse them.

#### Administering the PHQ-4 Form

Participants pointed out that it was difficult to interpret qualitative responses and translate them into a quantitative scale (PHQ-4 form). P1 told us that

At times, I was unable to gauge like an appropriate answer from his response so I wanted to interact with him more to see what that meant. I felt like I needed to pull more.

They further elaborated that they wanted to ask the VP more clarifying questions and have a more dynamic interaction to get the correct scale reading. P4 brought up that people usually don’t know how they feel, and it is MH workers’ jobs to be their advocate and interpret their response into a number and understand how to help them. Yet P4 expressed that

it really depends on the mental health professional right? Who is speaking to them like, what do they think that his, or her, or their response means, and translate all to a quantitative scale, which is difficult.

### Interacting With the VP

In this section, participants’ interaction with the VP will be reported, mainly their listening to the VP, observing the VP, and the flow of conversation with the VP. These qualitative insights help explain the strong interface quality score (mean 2.83, SD 1.59) attributed to the VP’s realistic body language, as well as the auditory challenges that impacted the Information Quality subscale (mean 3.76, SD 1.73).

#### Listening to the VP

Participants found that using a real person’s voice with an accent increased realism, immersion, and hence the usefulness of training for specific populations or communities. P1 remarked that the VP’s accent is very realistic, which reflects people in the community having different accents that might be more difficult to understand. P4 echoed this and felt like the voice was real because it had an accent. P4 elaborated that

If it was an automated voice I would have felt weird, that would have added to like the whole unrealness. Using a real person’s voice definitely helps… yeah, at one point… like it hit me that he wasn’t actually there. It was like just the two of us having a conversation.

Although P2 felt that the voice was impactful, they mentioned, “I wasn’t sure how to respond, because I couldn’t understand. I think his accent, and also like the volume was low.”

All participants experienced difficulty with hearing due to low volume and accent. However, this motivated them to listen more intently during the training, and they expressed that it was beneficial to train for real-life situations in which some people are more soft-spoken. P1 raised that “it trains me to hear, so it does serve a benefit” echoing P2, who noted “in a real-life situation, when someone is speaking softly or in a different accent, I would need to be listening more intently.” P4 reiterated “I really had to process, really listen, which is good, I was really listening.”

#### Observing the VP

Overall, participants highlighted that the body language of the VP was useful in training and increased realism, immersion, connection, and empathy. P1 mentioned that nonverbal cues were extremely helpful, for example, “the moving forward, the eyebrow expression.” P3 echoed the movement of the VP:

He actually felt kind of connected … there was a point where he kind of leaned back. I felt like it was important to just kind of lean in just a little bit to let him know that I heard and to let him know that he was okay. And I think that that’s so valuable and so important. It’s kind of given that empathetic piece.

Similarly, P4 also expressed that they felt empathy and connection toward the VP, highlighting that

when he leaned forward, I think maybe it was sort of his way of saying like, ‘Oh, this is between me and you, like when he was sharing whether he was feeling depressed.

P4 further inferred from the movement that “He was afraid that I would tell others. So I think that’s why he leaned forward.”

On the other hand, there were mixed views on the realism of the VP. P4 mentioned that the distance of the VP in the headset facilitated easier conversation with the VP. They highlighted,

I also like that he was actually sitting in front of me and he wasn’t too far or too close. I think the distance from where he was to me on the screen was helpful, because it was like it was just us two.

They also felt that the VP, being not too realistic and an animated avatar, made it easier to engage in conversation in a training setting. They added,

I’m like, would that actually be scarier for me, because it feels too real? I’ll probably be second guessing my responses, whereas here it felt like I was talking to someone my age, and I feel like, maybe that’s good.

On the other hand, P2 mentioned

I was more focused on how his eyes were not moving. It kind of gave me the uncanny valley feeling. I think I was so conscious of trying to maintain eye contact, I didn’t notice (the body language of the VP).

Hence, they requested more realism, specifically in the eye movements.

#### Flow of Conversation With the VP

Although participants found that the pauses were good in giving space for validation and mimicked what a conversation would be like in the field, more interactivity in responses was desired from the VP. P2 felt that the pauses gave them a chance to acknowledge how difficult the conversation was and recognized that people would appreciate the space and pauses for their feelings to be validated. However, P2 highlighted that

I couldn’t understand some of his responses. I don’t know if they were realistic… After I validated him, all he did was grunt. He never actually said it was hard, so I guess I had to be picking up on that, right? But yeah, I think he just didn’t interact that much.

P3 also echoed, “I was really interested in him elaborating more of what he was experiencing, and then reassuring him that it was okay.”

## Discussion

### Overview

Our findings indicate that the AR simulation demonstrated above-average usability, as reflected in PSSUQ scores and corroborated by qualitative accounts of realism, immersion, and empathetic engagement with the VP. Participants perceived the tool as a promising complement to existing training workflows, and the usability data informed a set of design recommendations organized across four domains: environmental context, training structure, VP behavioral realism, and technical hardware considerations (Multimedia Appendix 7).

To our knowledge, this is among the first studies to investigate the potential of using AR, rather than VR or desktop-based simulation, for training lay MH task-sharing workers with VP simulation. The majority of existing simulation research targets credentialed clinicians or nursing students in controlled settings; our study extends this work to community-based task-sharers who provide frontline MH support in underresourced communities [9,10,39-42]. Furthermore, the tool was co-designed through participatory methods with stakeholders from the HSI, ensuring that the training content and interaction design reflect the cultural and contextual realities of the population served [67,68].

### Principal Results

#### Current Challenges in MH Training

Traditional MH training often grapples with challenges in content simplification due to lack of resources [19,20]. Role-playing has often been used as a method to help trainees develop critical skills such as empathy, communication, and problem-solving [87]. While it is an effective way to simulate real-life scenarios and practice responses, it also presents challenges such as ensuring that role-plays are realistic and emotionally safe for participants, thus requiring skilled facilitators and significant preparation [87]. Echoing the literature, participants mentioned role-play in current training mostly being done between peers. Acting out more challenging scenarios, such as schizophrenia symptoms, was difficult to accurately role-play and might even be left out as peers were not trained actors. Current training often did not use trained actors as providers would require resources like time and money which they mostly do not have [88]. Our findings suggest that adding AR to the current workflow could increase more representative and effective role-play training.

Additionally, trainees may have theoretical knowledge but may require more intensive training in more nuanced practical skills such as reflecting on and interpreting people’s feelings and responses [89]. Participants emphasized the struggle of adapting theoretical knowledge to practice with current training methods. They expressed that theoretical knowledge is limited in its effectiveness, and they ultimately had to learn the ropes on the job and develop their own style of counseling. In addition, participants expressed difficulty when interpreting qualitative responses, translating them into a quantitative scale, and identifying how to help people due to individual subjectivity, reflecting their adaptability challenges with current traditional training. Notably, participants found that the AR training simulation allowed them to practice in a safe and comfortable environment where mistakes were acceptable and did not have detrimental consequences.

A systematic review found that racial and ethnic minority populations faced cultural MH stigma, which led to greater barriers to MH services and lack of trust [90]. Building trust between providers and patients in MH services is important to promote and maintain treatment and engagement [91]. Similarly, participants emphasized that building trust with patients was challenging but important. This type of engagement is difficult to train using traditional methods yet crucial for authentic patient relationships.

#### Enhancing Training Quality

An integrative review found that realism and debrief were important in MH simulation training and improved trainees’ engagement and in bridging the gap between theory and practice [92]. Our study’s results highlight that the realism of the AR simulation and the quality of the animations led participants to feel empathy and connection toward the VP. Participants emphasized that they felt like they (the VP and participant) were the only ones in the room, indicating that the AR simulation was successful in immersing participants into a real-life scenario of conversing with a community member. The VP’s voice had an accent and was realistic, reflecting the population they would be interacting with, further allowing participants to feel like they were talking to a real person and practice communication. The VP’s animation and body language allowed trainees to further empathize with the VP and practice building trust. Through MH training simulation with SPs, trainees could gain empathy, compassion, and confidence, benefiting the people they serve [93]. The AR simulation was realistic, immersive, and sparked empathetic connections, thereby allowing trainees an environment to practice engaging and building trust in a realistic and effective manner.

Additionally, participants highlighted that AR simulation can better depict challenging scenarios, especially specific DSM categories like schizophrenia, depression, and anxiety. They emphasized that a standardized role-play training that best accurately depicts symptoms, rather than peers acting out the symptoms, would be extremely useful for practice. As such, having realistic practice in AR before real-life interactions can build provider efficacy and better care for patients. This kind of applied training is crucial for effective care by providers and MH task-sharers, with studies showing that using AR or VR to practice in a safe environment enhances knowledge and transferable skills, leading to the transfer of knowledge and skills in clinical practice [94]. Thus, this AR-assisted training tool could bridge the gap in training accuracy and enrich current traditional role-playing training.

#### The Role of Realism and Immersion in AR Training

##### Overview

Studies have shown that higher realism in immersive virtual experiences leads to a more positive user experience, highlighting that degrees of realism affect the extent of immersion and thus the training’s effectiveness [95]. When such training elicits emotional responses similar to those experienced in real-life situations, empathy is found to be nurtured in trainees [36]. Thus, it is important to create a realistic training experience for trainees to develop empathy and immerse fully. In this section, the factors that contribute to realism include the VP’s body language, voice, distance from the trainee, appearance, eye movements, and dialogue.

##### Body Language

Nonverbal cues such as facial and body movements have been shown to facilitate connection with VPs [96]. Participants in our study empathized with the VP and wanted to reassure, validate, and comfort the VP when they perceived distress through the VP’s body language. We recommend designing VPs with realistic nonverbal cues to enhance immersion and empathy (Multimedia Appendix 7).

##### Voice

Virtual assets’ realism and appropriate contextualization enhance one’s perceptual skills, which are essential for developing sensemaking abilities when using AR [97]. Our study’s VP had a real person’s voice with an accent, and participants pointed out that this was very useful for training their listening skills, especially for specific populations or communities they may be training for. They added that the headset’s technical limitation, that led to the soft volume, was actually useful in training them to listen more attentively to soft-spoken people. These findings emphasize that voice realism in the VP is useful and crucial for participants to hone their sensemaking skills in MH training. Using a representative real person’s voice and varying volume levels can train attentive listening skills (Multimedia Appendix 7).

##### Distance From Trainee

The results highlight that the distance of the VP from the participants was realistic enough, not too close or too far, and that this facilitated easier engagement and immersion in the training. This corroborates with literature findings that training systems which are context-specific enhance learning [97]. Placing the VP at a therapeutically informed comfortable distance enhances immersion (Multimedia Appendix 7) [98].

##### Appearance

Our findings, also supported with literature, suggest that representing the VP as an animated 3D avatar instead of making the VP too human-like helped in avoiding the uncanny valley effect; this is the discomfort or eeriness experienced when digital avatars closely resemble human beings but fall short in certain aspects of realism [99,100]. Balancing avatar realism to avoid the uncanny valley effect is recommended (Multimedia Appendix 7).

##### Eye Movements

One participant perceived the VP’s eyes as staying still, and it reduced their ability to notice body language cues that were programmed into the training module and broke the immersion for them. The perception of the eyes not moving, even though they were programmed to move, contributed to them experiencing the uncanny valley effect. This highlights the importance of making the eye movements more realistic and obvious to ensure that the uncanny valley effect is minimized and to increase immersion, especially when gaze-tracking is correlated to trainees’ assessment skills [97]. Realistic and obvious eye movements are essential for maintaining immersion (Multimedia Appendix 7).

##### Dialogue

Although pauses in dialogue were good in giving space for validation and mimicked a real conversation in the field, more interactivity in the VP’s responses was desired by participants. In addition, one of the participants felt like the responses were lacking in realism; for example, the VP grunting as a response. These findings align with evidence that dynamic, contextually responsive VP dialogue is crucial to perceived realism in simulation-based training [21,97]. More interactive, dynamic VP responses would enhance conversational realism (Multimedia Appendix 7).

Immersion was also affected by the structure of the training. This included the use of prompts, which have been found to be useful for guiding learning [97]. Although the prompts were useful in guiding participants on how to respond when they were unsure, navigating between two types of prompts confused users and reduced immersion. Switching between a general prompt and a specific word-for-word prompt (Figure 7), paying attention to the VP’s body language and dialogue, and asking the questions from the PHQ-4 form broke the flow of immersion and caused confusion in participants at times. Clearly distinguishing prompt types and gradually transitioning from guided to unguided interactions would improve training flow (Multimedia Appendix 7).

### Overall Usability and Acceptability of AR Tool

Our findings show that the AR-assisted training tool is useful for MH training and has the potential to scale to different MH contexts, especially more complex scenarios. Qualitative results highlight the overwhelming positive sentiments toward the AR tool and its promising potential in successfully filling the gaps of current training workflows. The overall PSSUQ scores indicated above-average usability, consistent with patterns reported in comparable simulation-based training evaluations [92,101]. The interface quality subscale performed particularly well, suggesting that the AR tool’s interface met user expectations for functionality and pleasantness, an outcome that aligns with findings that higher-fidelity immersive environments tend to yield stronger user acceptance [36,95]. High reported desire to use the system frequently further supports the tool’s acceptability as a complement to existing training workflows.

The main challenges of integrating this tool into the wider MH training community are the technology learning curve and hardware limitations. Two out of the three first-time users of AR and VR headsets experienced difficulty at the start. Although they expressed that they were overwhelmed by all the different things to pay attention to, they also highlighted that with more practice, they would adjust to the training and benefit from it. This is a positive outcome since AR and VR headsets are known to have a steep learning curve [101]. The two participants who raised this concern were in the 35- to 40- and 56- to 60-year-old range, respectively, which may indicate that the older demographic, who tend to have a lower technology learnability rate, could find this AR tool to have a manageable learning curve [102]. We recommend keeping this challenge in mind and working on building a seamless onboarding process when introducing a similar training tool.

Another challenge for integration is the headset’s hardware limitations, including the headset falling down due to a missing accessory pack for supporting a range of nose bridges, the headset heating up, and low audibility even on the headset’s maximum volume. This was an internal software issue that could be fixed in hindsight. The headset falling down affected the PSSUQ scores, particularly as this discomfort led to breaking participants’ immersion. A study using similar AR HMDs also mentioned that issues such as user discomfort and technical limitations of HMDs need to be addressed before widespread adoption in the health care industry [103]. Thus, these challenges must be addressed for a successful implementation of this tool. We recommend addressing hardware fit, comfort, and cost challenges; integrating familiar training workflows; incorporating personalized feedback mechanisms; and enabling VP response replay (Multimedia Appendix 7).

### Limitations

This study has several limitations that should be considered when interpreting the findings. First, as an exploratory study, the work reported here involved a limited number of participants, making it difficult to draw generalizable conclusions. Due to the small sample size, a descriptive analysis was used instead of a test of significance. The selection of the “N/A” option affected the quantitative results. To minimize this, an additional instruction could have been given to participants before they filled out the quantitative survey to remind them that if they did not understand the question, they could clarify with researchers. Due to the attrition of one participant (P5) and the small pilot sample size, thematic saturation was not reached; however, the data collected from the remaining four participants provided consistent insights into the tool’s usability. This study was intended to understand usability and user perceptions rather than to evaluate training effectiveness or learning outcomes. The promising results indicate the need for future studies, such as collecting quantitative data from a larger sample size and measuring trainees’ actual performance after AR-assisted training.

Second, the hardware challenges might have affected the data. Participant satisfaction with AR training has been shown to vary depending on technical factors [104]. Comfort issues such as overheating from low battery life, accommodating glasses wearers (which caused a participant to drop out of the usability test), and the lack of an adaptive fit for certain nose bridges and head sizes could not be overlooked. The hardware costs also presented challenges for the scalability of being widely used in the community.

Third, the study was conducted in a single site; hence, our results may lack transferability to other settings with different parameters, which is an especially important factor to consider for educational learning interventions [105]. The investigation was also context-specific, which may not be generalizable to screening for other MH conditions. Using more in-depth scales to test realism and immersion and the relationship it has with effectiveness in a different experiment would also further enhance this study.

### Conclusions

This pilot study provides promising preliminary evidence that AR-based VP simulation is a usable and engaging training modality for MH task-sharers. The study is among the first to apply AR glasses with VP simulation to this population, filling the gaps of past studies that have predominantly used VR or desktop-based systems with credentialed clinicians and nursing students. This pilot study brings preliminary empirical usability evidence from a validated instrument (PSSUQ) demonstrating above-average usability, and a set of interaction design recommendations spanning environmental context, training structure, VP behavioral realism, and technical hardware considerations. These findings show promising results that AR-based simulation could enable MH task-sharers in underresourced community settings to access realistic, culturally tailored training practice at a lower financial, labor, and time cost, directly addressing the resource constraints of current training approaches. Future research should explore cost-effective hardware alternatives that address comfort and accessibility challenges, increase sample sizes for inferential analysis, compare AR-assisted training simulations with traditional methods, and extend the tool to additional MH conditions for greater generalizability and scalability.

## Supplementary material

10.2196/80711 Multimedia Appendix 1 ARATT demo.

10.2196/80711 Multimedia Appendix 2 Screening Scenario script.

10.2196/80711 Multimedia Appendix 3 Semistructured interview questions.

10.2196/80711 Multimedia Appendix 4 Qualtrics questionnaire.

10.2196/80711 Multimedia Appendix 5 Summary of key themes.

10.2196/80711 Multimedia Appendix 6 Quantitative responses table.

10.2196/80711 Multimedia Appendix 7 Summary of design recommendations.

## Abbreviations

APA: American Psychiatric Association
AR: augmented reality
CIMH: Center for Innovation in Mental Health
DSM: *Diagnostic and Statistical Manual of Mental Disorders*
HMD: head-mounted display
HSI: Harlem Strong Initiative
INFOQUAL: information quality
INTERQUAL: interface quality
MH: mental health
MR: mixed reality
NDA: National Institute of Mental Health Data Archive
PHQ-4: Patient Health Questionnaire-4
PSSUQ: Post-Study System Usability Questionnaire
SP: standardized patient
SYSUSE: system usefulness
VP: virtual patient
VR: virtual reality
