# Supplementary material for: Augmented Reality–Assisted Training Tool for Mental Health Task-Sharers: Pilot Mixed Methods Usability Study
Source: JMIR XR Spat Comput. 2026 Jun 25;3:e80711. doi: 10.2196/80711 (PMC13297265; doi:10.2196/80711)
Supplement: Multimedia Appendix 2 [file xr-v3-e80711-s002.pdf]

States

| # | Prompt | Correct Dialogue | Consumer Response | Animation Clip |
|---|---|---|---|---|
| 1 | Introduce yourself and request the consumer's name. | What's your name? | Alvaro. | Sitting Idle |
| 2 | Express enthusiasm about meeting. Ask an open-ended question about the consumer's wellbeing. | Nice to meet you. Can you tell me a little bit about what's been going on for you? | I guess . . . I don't really know where to start. | Knee Tap |
| 3 | Express acceptance of the consumer. Explain the purpose of screening. | That's okay. One thing we use here is a screening tool, which has just four questions about how you've been feeling in the past two weeks. Answering these questions can help us understand how you are doing and how we can best support you. | Okay. | Sitting Idle |
| 4 | Ask about the consumer's current feelings. | Is it feeling a little weird to talk about this? | Yeah I guess. | Sitting Idle |
| 5 | Normalize disclosure of distress during the screening process. Reiterate the purpose of screening. | It's really common to feel a little awkward during this part! And answering these questions will help me and our team better understand how we can help, okay? | Okay. | Sitting Pose |
| 6 | Ask screening question 1 from PHQ-4. | How often would you say you've been nervous, anxious, or on edge? Would you say not at all, several days, more than half the days, or nearly every day? | On edge? Not really. | Sitting Pose |
| 7 | Summarize the consumer's question 1 response. Ask screening question 2 from PHQ-4. | Okay, so not at all. And again, thinking about the last two weeks, how often have you felt you weren't able to stop or control your worrying? Would you say not at all, several days, more than half the days, or nearly every day? | I don't really worry that much. | Sitting Pose |
| 8 | Clarify the consumer's question 2 response. | Okay, so how often would you say that you do worry? | Sometimes. | Sitting Idle |
| 9 | Ask screening question 2 again. | Okay, so would you say, like several days in the last two weeks? More than half of the days? | I guess. I would say. like yeah. several days. | Sitting Idle |
| 10 | Ask screening question 3 from PHQ-4. | Okay, thanks for letting me know about that. And how often in the last two weeks have you been feeling down, depressed, or hopeless? | . . . | Sitting Pose |
| 11 | Give the consumer time to process. | . . . | Sorry. can you repeat the question? | Sitting Pose |
| 12 | Repeat PHQ-4 question 3. | Sure, no problem. How often in the last two weeks have you been feeling down, depressed, or hopeless? Would you say not at all, several days, more than half the days, or nearly every day? | What's going to happen if I say every day? | Sitting Idle |
| 13 | Encourage the consumer to share how they feel. | We're just going to think together about how we can support you. | Okay. I guess. a lot of the time. | Sitting Idle |
| 14 | Clarify the consumer's response. | So, every day? | Yeah. I guess. You're not going to tell nobody. though. right? | Sitting Idle |
| 15 | Reassure the consumer that their information will be kept confidential unless doing so poses an immediate threat to their own or someone else's life. | No, we are going to keep all of the parts of this conversation private between our team to support you unless you say that you're going to harm yourself or someone else. | Okay. | Sitting Pose |
| 16 | Ask if the consumer has any questions. | Do you have any other questions about that? | No. | Sitting Pose |
| 17 | Ask screening question 4 from PHQ-4. | Okay. well just let me know if you have other questions. I have one more for you: How often in the last two weeks have you been feeling less interested in doing the things you normally enjoy? Would you say not at all, several days, more than half the days, or nearly every day? | How often did I not enjoy . . .? | Knee Tap |
| 18 | Repeat the question. | Yeah, how often did you not enjoy things that you usually like? | Yo. it is terrible out here sometimes. How can you enjoy anything? | Knee Tap |
| 19 | Empathize (validate, affirm, normalize, or reassure) the consumer and return to the screener. | Oh yeah, it is brutal out there right now. But separately from the weather, how often in the last two weeks have you been feeling less interested in doing the things you normally enjoy? Would you say not at all, several days, more than half the days, or nearly every day? | Hmm. This question I don't know. I don't know when the last time I enjoyed the stuff I used to enjoy was. | Sitting Pose |
| 20 | Summarize back what you heard the consumer say. | Okay. So what I'm hearing overall is that it's harder for you to enjoy things lately, and you've been feeling kinda down, and sometimes you're worrying about stuff. | . . . | Sitting Pose |

| | | | | |
|---|---|---|---|---|
| **21** | Express that the program may help. | Yeah. so this has been a little bit of a tougher time for you. This indicates to us that you may benefit from some more support through our program. | You think I'm crazy and you're going to throw me in the loony bin? | Sitting Idle |
| **22** | Reassure the consumer about how the program will provide support. | No, we think you're telling us you're stressed out, and we want to talk to you about how you can manage that stress differently so that you can feel better. | I've been messing up. the ways that I'm managing it. huh? | Sitting Pose |
| **23** | Reassure the consumer that it's not their fault. | No, it's not something that most people learn, so it's not your fault that you didn't learn it. It would be great if they taught this stuff in school. | Yeah. I guess. | Sitting Idle |
| **24** | Thank the consumer for talking with you. | Well thanks so much for talking to me about this. I know it's not always easy. | Yeah. I wasn't looking forward to it. but I guess it wasn't so bad. | Sitting Idle |
