# Supplementary material for: Augmented Reality–Assisted Training Tool for Mental Health Task-Sharers: Pilot Mixed Methods Usability Study
Source: JMIR XR Spat Comput. 2026 Jun 25;3:e80711. doi: 10.2196/80711 (PMC13297265; doi:10.2196/80711)
Supplement: Multimedia Appendix 3 [file xr-v3-e80711-s003.pdf]

Semi Structured Interview Questions

<u>Pre-Study Interview</u>

A. Please introduce yourself, and say what your experience with CUNY's mental health skills module. Also please tell us about your role in the community and a little about your training and experience.
B. Are there any challenges you face in your day-to-day interactions with community members that might be under distress, sad/down, anxious, or dealing with other mental challenges? How do you handle these situations currently?

<u>During Testing</u>

- Describe what you see.
    - Do you see a character in front of you with a prompt card?
    - Show us how you would proceed.

  [Start AR headset voice listening]

- [when a "Try this" card comes up]
    - Show us how you would proceed.

- Continue going through this exercise while thinking out loud. We may chime in with questions at certain points.
    - [If participant does something unexpected, some questions researcher can ask:]
        - What did you expect to happen?
        - Is there anything in particular you would like to see at this point?

- [body language pause] What do you notice about the character's response?
    - Did you notice when the character <u>turns away / looks down / shrugs</u>?
    - For your work, would practicing noticing people's body language be helpful?
    - Is there anything in particular you would like to see at this point?

- [PHQ4 ended pause]
    - For your work, would practicing asking the PHQ4 be helpful?
    - Is there anything in particular you would like to see at this point?

<u>Post-Study Interview</u>

Keeping in mind how you would like to go through mental health training and its current challenges, can you tell us:

1. What aspects of the prototype worked?
2. What aspects of the prototype didn't work?
3. Would this be something you find useful when interacting with community members that might be under distress, sad/down, anxious, or dealing with other mental challenges?
4. Are there changes that you might like to see? E.g. additional features? Or things that could be made easier?
5. Is there anything else you think we should know that is relevant to the tool we have just evaluated?
