# Supplementary material for: Augmented Reality–Assisted Training Tool for Mental Health Task-Sharers: Pilot Mixed Methods Usability Study
Source: JMIR XR Spat Comput. 2026 Jun 25;3:e80711. doi: 10.2196/80711 (PMC13297265; doi:10.2196/80711)
Supplement: Multimedia Appendix 4 [file xr-v3-e80711-s004.pdf]

Usability Scale

On a scale between Strongly Agree to Strongly Disagree, please rate the following statements:

	1 (Strongly agree)	2	3	4	5	6	7 (Strongly disagree)	N/A
1. Overall, I am satisfied with how easy it is to use this system.	○	○	○	○	○	○	○	○
2. It was simple to use this system.	○	○	○	○	○	○	○	○
3. I was able to complete the tasks and scenarios quickly using this system.	○	○	○	○	○	○	○	○
4. I felt comfortable using this system.	○	○	○	○	○	○	○	○
5. It was easy to learn to use this system.	○	○	○	○	○	○	○	○
6. I believe I could become productive quickly using this system.	○	○	○	○	○	○	○	○
7. The system gave error messages that clearly told me how to fix problems.	○	○	○	○	○	○	○	○
8. Whenever I made a mistake using the system, I could recover easily and quickly.	○	○	○	○	○	○	○	○
9. The information (such as online help, on-screen messages, and other documentation) provided with this system was clear.	○	○	○	○	○	○	○	○
10. It was easy to find the information I needed.	○	○	○	○	○	○	○	○

	1 (Strongly agree)	2	3	4	5	6	7 (Strongly disagree)	N/A
11. The information was effective in helping me complete the tasks and scenarios.	○	○	○	○	○	○	○	○
12. The organization of information on the system screens was clear.	○	○	○	○	○	○	○	○
13. The interface of this system was pleasant	○	○	○	○	○	○	○	○
14. I liked using the interface of this system.	○	○	○	○	○	○	○	○
15. This system has all the functions and capabilities I expect it to have.	○	○	○	○	○	○	○	○
16. I think that I would like to use this system frequently.	○	○	○	○	○	○	○	○
17. Overall, I am satisfied with this system.	○	○	○	○	○	○	○	○

Demographics

1. What is your age?

- ○ Below 16 years old
- ○ 16 - 20 years old
- ○ 21 - 25 years old
- ○ 26 - 30 years old
- ○ 31 - 35 years old
- ○ 35 - 40 years old
- ○ 41 - 45 years old
- ○ 46 - 50 years old
- ○ 51 - 55 years old
- ○ 56 - 60 years old

- 61 - 65 years old
- 66 - 70 years old
- 70+ years old
- Prefer not to say

2. What gender do you identify as?

- Male
- Female
- ________ Other ,specify below
- Prefer not to say

3. Please specify your ethnicity. (Tick all that apply)

- Caucasian
- African-American
- Latino or Hispanic
- Asian
- Native American
- Native Hawaiian or Pacific Islander
- ________ Other, specify below
- Prefer not to say

What is the highest degree or level of education you have completed?

- High School
- Bachelor's Degree
- Master's Degree
- Ph.D.
- Trade School
- ________ Other, specify below
- Prefer not to say

How many times have you used an Augmented Reality headset or glasses before this (devices similar to what was used for the study)?

- ○ 0
- ○ 1
- ○ 2
- ○ 3
- ○ [ ] Other, specify below

Powered by Qualtrics
