# Supplementary material for: Augmented Reality–Assisted Training Tool for Mental Health Task-Sharers: Pilot Mixed Methods Usability Study
Source: JMIR XR Spat Comput. 2026 Jun 25;3:e80711. doi: 10.2196/80711 (PMC13297265; doi:10.2196/80711)
Supplement: Multimedia Appendix 5 [file xr-v3-e80711-s005.pdf]

Domains	Insight	Representative Statements
Pre-Study Interviews		
Trust	Building trust with clients is challenging but important	"we definitely use a lot of open ended questions. We try to draw out the student, and, unlike a regular advising session that you may experience as an undergrad where you go once a semester. This was part of an ongoing program, so I met with the student once a week. So there was already rapport built there"<br><br>"Trust is a huge factor in getting community members to know that you're there for them, that you're concerned about their well-being. You'd like to maybe introduce them to some practices that they may not be aware of or might not understand fully and let them know that it's okay."<br><br>"I might tend to go a little bit further than intended for whatever the session is supposed to be, as more of a listening ear type thing, and just try to engage in trying to get more information from them that I might not necessarily use."
Application Challenges	Difficulty in adapting theoretical knowledge to practice in current training	"because every encounter is different, it's kind of very foundational. You have to develop your own way of counseling."<br><br>"when you come into actually working in the field, it's a little different. You might be in a setting where you're dealing with things that are the least favorable settings right."
Current Training	Current training included role play and usually did not include difficult scenarios	"covered the DSM. We did a lot of role play"<br><br>"normally, when we do role plays in class, nobody goes down that road of re-enacting more challenging scenarios like schizophrenia, people tend to present the lighter stuff that might not be entirely accurate."
	Content of client sessions	"Well, you know, when I interact with students, essentially the student would be sitting here. I'd have the computer monitor here. I have my case notes, so I'd be typing while talking to the student, but after the first few sessions it feels natural. So it's similar, probably, through this training wheels approach, or even having read the prompts prior to sitting down, it would have probably been more like a natural conversation than mechanical, you know."<br><br>"Most of the sessions that I work with were 20 min sessions. So if I had 20 min to gauge how a person's week went, you know, to go through anything larger than like 5 questions about their moves and their temperament."
Think-aloud Insights		
Use of physical headset	Fit, heating up broke immersion	"it broke the immersion to me. I think it would feel more like I'm talking to someone if the headset didn't keep falling, I was distracted by it."<br><br>"it didn't fit me... like it's just slipping down everywhere. My arms were like sore from holding it up because they're heavy, too. But yeah, I think I was a little bit distracted by how like it kept looking down. So I like I think it could have gone better if it fit better on my face, because I was so distracted by like, just like the glasses on my face, but only because it was slipping down.<br><br>"So the headset is heating up which makes it hard for me to hold on my face."
Delivery of content	Volume was too low, content might not be clear	"It was a little hard to hear him. I also feel bad, because I didn't get a name. I didn't hear it. Okay, yeah, I didn't hear them say a name so that might have just been during the time when the volume wasn't working properly. Because I wanted to address him with his name."<br><br>"Like the sound was really low, so I really had to like process, really listen, which is good, I guess, like I was like really listening. But that was also because yeah, it was very low."<br><br>"I feel kind of bad, but it was like a little bit soft for me even on the maximum."<br><br>"…it was a bit mumbled as well."
Post-Intervention Interviews		
Experiential	Positive sentiments towards the training module	"it's a really good training module and the prompts were very helpful in guiding".<br><br>"I thought it was cool. This is my first time I did. It was really cool."<br><br>"I think maybe having specific augmented reality trainings for specific DSM categories would be very helpful … like depression and anxiety … if it's tailored towards, you know, something that may be heavier, that would be interesting."
	Realism of the simulation allowed for empathy with the virtual patient	"it kind of forces you to empathize. It forced me to look at the AI almost like a real person, like, this person has an accent."<br><br>"it was like just the two of us having a conversation."<br><br>"It begins to feel like the person, the quality of the animation allowed me to connect with them."

Experiential Satisfaction	Potential usefulness of simulation in other contexts	"So I think maybe having specific augmented reality trainings for specific DSM categories would be very helpful. It'd be much better to do a role play with somebody who let's say presents as schizophrenic through augmented reality than to have to do that cold turkey in real life, you know. And normally, when we do role plays in class, nobody goes down that road, you know people present the lighter stuff that's on the top. "<br><br>"...mostly all the mental health conditions that we need! I find ourselves engage in… and just knowing, or having the knowledge I think that would be important."
	Struggles for first time AR/VR headset users	"This was my first VR experience with this type of thing. So perhaps you know, if this was my second or third, I would know, like, okay, you know I can read the prompt and look at the the patient. The prompts to me were just a little bit more challenging, because then I didn't know whether to focus on the prompts or to focus on the patient."<br><br>"So, because this was new, and I'm trying to look at everything and navigate at the same time. So you have the prompts. You have the client. You have the questionnaire. And you have the volume. And then you have the potential language barrier just a bit. And I think volume kind of was a disability for me, because I couldn't even hear him clearly. I was not really sure of what he was saying, so I wouldn't really grab or gravitate towards bits and pieces of what I felt like, he was saying. And then also look at the body language to see what was conveyed to make a determination and how, you know, what it intended to express itself."
Navigating the training	Guided prompts were effective but switching between two prompts caused confusion	“I'm not sure if I'm like you know, helping or hurting. So that's why having the prompts helped. It's like semantics also have impact on how you affect the client..”<br><br>“…at times I was really lost as to what to say, but also because I didn’t hear him so well. That kind of made it more helpful for the prompt, because I was like, okay, this is what I say now.”<br><br>“when it's the “Try this” prompt, I'm like kind of fed what to say, but then there’s the other prompt where I kind of have to think on my feet. Switching back and forth was kind of confusing for me.”<br><br>"trying to figure out which to do when between the two may have taken away from the experience of just looking at the avatar and be like, okay, you know what's going on. What are their body languages, whatever it may be, tone of voice, things of that nature."<br><br>"I didn't really get a chance to do that, because I was just trying to follow the prompt and trying to hear. And so the prompts might have been a little limiting at times. It’s just trying to coordinate everything. So the reading of the the question, the statement, listening for what it was he's saying, and then also paying attention to his body language, and you know just trying to hear."
	Administering the PHQ-4 form was difficult to adapt qualitative responses to quantitative scale	“At times, I was unable to gauge like an appropriate answer from his response so I wanted to interact with him more to see what that meant. I felt like I needed to pull more.”<br><br>“it really depends on the mental health professional right? Who is speaking to them like, what do they think that his, or her, or their response means, and translate all to a quantitative scale, which is difficult.”
	Listening to the virtual patient: accent increased realism, immersion and empathy	"At first I thought maybe I wasn't hearing the name correctly, but I think having the patient with an accent, it’s very realistic like that, you know, and it is hard to understand. Sometimes when people who have names you're not used to hearing speak, and then you have to try to pull that out. So working in the neighborhood, or a community where you have patients like that, and they speak very fast or speak very low."<br><br>"The voice I feel like the voice was real enough. I feel like, because it it was an accent."<br><br>“If it was an automated voice I would have felt weird, that would have added to like the whole unrealness. Using a real person's voice definitely helps… yeah, at one point I was just kind of… like it hit me that he wasn't actually there. It was like just the two of us having a conversation.”<br><br>”I wasn't sure how to respond, because I couldn't understand. I think his accent, and also like the volume was low.”<br><br>“it trains me to hear, so it does serve a benefit”<br><br>“in a real life situation, when someone is speaking softly or in a different accent, I would need to be listening more intently.”<br><br>“I really had to process, really listen, which is good, I was really listening.”

Interacting with virtual patient	Observing the virtual patient: body language increased immersion and empathy, realism of virtual avatar affects immersion	"the moving forward, the eyebrow expression. I've noticed, and I think those non verbal queues are extremely helpful."<br><br>“He actually felt kind of connected … there was a point where he kind of leaned back. I felt like it was important to just kind of lean in just a little bit to let him know that I heard and to let him know that he was okay. And I think that that's so valuable and so important. It's kind of given that empathetic piece.”<br><br>“when he leaned forward, I think maybe it was sort of his way of saying like ‘Oh, this is between me and you, like when he was sharing whether he was feeling depressed.’”<br><br>“He was afraid that I would tell others. So I think that's why he leaned forward.”<br><br>“I also like that he was actually sitting in front of me and he wasn't too far or too close. I think the distance from where he was to me on the screen was helpful, because it was like it was just us two.”<br><br>“I'm like, would that actually be scarier for me, because it feels too real? I’ll probably be second guessing my responses, whereas here it felt like I was talking to someone my age, and I feel like, maybe that’s good.”<br><br>“I was more focused on how his eyes were not moving. It kind of gave me the uncanny valley feeling. I think I was so conscious of trying to maintain eye contact, I didn't notice (the body language of the virtual patient).”
	Flow of conversation with virtual patient: pauses were effective but wanted more interaction and dynamism from virtual patient	"I like the pauses where you were giving the interviewer (me) a chance to like acknowledge that it's hard to talk about this stuff. Yeah, I think like as a patient, I would feel valued if someone were to say to me like, oh, it's okay, what you're going through, like that was good."<br><br>“I couldn't understand some of his responses. I don't know if they were realistic … after I validated him, all he did was grunt. He never actually said it was hard, so I guess I had to be picking up on that, right? But yeah, I think he just didn't interact that much.”<br><br>“I was really interested in him elaborating more of what he was experiencing, and then reassuring him that it was okay.”
