# Supplementary material for: Augmented Reality–Assisted Training Tool for Mental Health Task-Sharers: Pilot Mixed Methods Usability Study
Source: JMIR XR Spat Comput. 2026 Jun 25;3:e80711. doi: 10.2196/80711 (PMC13297265; doi:10.2196/80711)
Supplement: Multimedia Appendix 6 [file xr-v3-e80711-s006.pdf]

Appendix: Quantitative Responses Table

Question	1 (Strongly Agree)	2	3	4	5	6	7 Strongly Disagree	N/A
**1. Overall, I am satisfied with how easy it is to use this system.**		2		1		1		
**2. It was simple to use this system**		2		1		1		
**3. I was able to complete the tasks and scenarios quickly using this system.**		2	1			1		
**4. I felt comfortable using this system.**		1	1		1	1		
**5. It was easy to learn to use this system.**		2			1	1		
**6. I believe I could become productive quickly using this system.**	2			1	1			
**7. The system gave error messages that clearly told me how to fix problems.**			1		1	1		1
**8. Whenever I made a mistake using the system, I could recover easily and quickly.**		1	1		1	1		
**9. The information (such as online help, on-screen messages, and other documentation) provided with this system was clear.**			1		1	1		1
**10. It was easy to find the information I needed.**		2			1	1		
**11. The information was effective in helping me complete the tasks and scenarios.**	1	1	1		1			
**12. The organization of information on the system screens was clear.**	1	1	1		1			
**13. The interface of this system was pleasant.**	1	1	1		1			
**14. I liked using the interface of this system.**	2		1		1			
**15. This system has all the functions and capabilities I expect it to have.**		2		1	1			
**16. I think that I would like to use this system frequently.**	1	1		1	1			
**17. Overall, I am satisfied with this system.**	1	1		1	1			
