# Supplementary material for: Augmented Reality–Assisted Training Tool for Mental Health Task-Sharers: Pilot Mixed Methods Usability Study
Source: JMIR XR Spat Comput. 2026 Jun 25;3:e80711. doi: 10.2196/80711 (PMC13297265; doi:10.2196/80711)
Supplement: Multimedia Appendix 7 [file xr-v3-e80711-s007.pdf]

Summary of Design Recommendations

	Recommendation	More Details
**Context**	Utilize AR for role-playing scenarios that are challenging to execute in traditional settings.	Expand content and simulation cases for specific DSM categories that are more complex like depression, anxiety and schizophrenia. Work with clinicians and experts to craft an accurate script for each simulation, taking into account body language behaviors, speech patterns, and prompts to guide trainees to respond. If resources are available, use an actor for voiceover and motion capture of the body and facial movements of the avatar.
**Body Language**	Ensure virtual patients are designed with realistic non-verbal cues that accurately portray patient symptoms	including facial and body movements, to increase realism, immersion, connection and empathy.
**Voice**	Use a real person's voice, representative of the target community, to enhance realism and engagement.	Including different accents will also increase the usefulness of the training. This should also be reflected respectively in the appearance of the virtual patient.
	Intentionally vary the volume of the virtual patient during part of the training.	This will help trainees to listen more intently to people who are more soft spoken, giving them the opportunity to practice engaging with them. Give participants agency to repeat hearing responses if they are unable to understand or hear clearly.
**Distance from trainee**	Include a feature to place the virtual patient at a comfortable distance from the trainee to mimic real-life situations and thus increase immersion.	The comfortable distance can be deduced from the therapeutic distance framework where the distance is correlated to the patient's attachment style (Egozi et. al., 2021)
**Appearance**	Ensure that the virtual patient is not too human-like.	Balancing the realism in the representation of the virtual patient is crucial. It should not be too realistic and still embody some cartoonish and animated aspect to avoid the uncanny valley effect.
**Eye Movements**	Focus on including realistic body language animations, especially in making eye movements realistic and obvious.	Engaging in conversation often involves a great degree of eye contact, hence making sure the eyes blink and move realistically is crucial in making the experience realistic and avoiding the uncanny valley effect.

**Dialogue**	Include interactivity and realism in virtual patient's responses.	This can come in a form of a more dynamic conversational AI. For example, when clarifying questions are asked and responses have been replayed once, the virtual patient will respond in a different way, reflecting a more realistic conversation rather than a "test question" you have to answer correctly.
**Structure of training**	Distinguish prompts clearly.	One way this can be done is having the generic prompts as the default prompt and when the trainee is unsure how to proceed, they can click a button that brings in the word-for-word prompt in a different UI to ensure there is no confusion.
	Training wheels approach to build provider efficacy.	A gradual transition from guided prompts to unguided interactions as practice progresses was supported by the 2 participants who have experience in the field. This also mimics current workflows mentioned by participants whereby prompts or notes are used less as sessions increase with clients.
	Integrate features that reflect current training practices, enhancing familiarity and reducing the learning curve.	Designing training that reflects current mental models increases learning efficacy (Sushereba et al., 2021). For example, the ability to personalize the placement of prompts according to current workflow and flagging to supervisors for challenging cases.
	Incorporate features for personalized user feedback to enhance usability.	Implement end-of-session feedback mechanisms to allow for review and guide improvements. Lee et al. (2020) emphasizes how learners can identify flaws and improve skills and confidence in therapeutic communication through recording and reviewing communication between trainees and virtual patients. Simple feedback and scores could also efficiently motivate trainees' self-directed learning (Verkuyl et al., 2018).
	Offer features allowing trainees to replay client responses during training.	This helps to prevent a break in flow when they do not understand or when a technical glitch occurs, allowing for greater understanding and encouraging more profound engagement.
**Technical Considerations**	Address hardware challenges, like fit, comfort, volume, costs for scalability and sustainability.	Investigate alternatives to ensure more universal fit, especially for glasses wearers, evaluate the adaptability of the headset's nosepiece, and, if necessary, include accessories for supporting different nose bridges. Prepare a hardware checklist to ensure all relevant accessories, such as different nosepieces, are readily at hand. The headset should not be heating up to an uncomfortable level, and the volume needs to be audible. Look into cheaper options so that the training

		can be implemented on a large scale, and consider the long-term stability of the software and hardware.
